# Risk factors for common mental disorders in young refugees from Iran, Somalia and Syria to Sweden

**DOI:** 10.1093/pubmed/fdad034

**Published:** 2023-04-12

**Authors:** Lijun Yang, Ellenor Mittendorfer-Rutz, Ridwanul Amin, Magnus Helgesson

**Affiliations:** Department of Clinical Neuroscience, Division of Insurance Medicine, Karolinska Institutet, Stockholm SE-171 77, Sweden; Department of Clinical Neuroscience, Division of Insurance Medicine, Karolinska Institutet, Stockholm SE-171 77, Sweden; Department of Clinical Neuroscience, Division of Insurance Medicine, Karolinska Institutet, Stockholm SE-171 77, Sweden; Department of Clinical Neuroscience, Division of Insurance Medicine, Karolinska Institutet, Stockholm SE-171 77, Sweden

**Keywords:** common mental disorders, emigration and immigration, mental disorders, mental disorders, refugee, refugee minor, Sweden

## Abstract

**Background:**

Our primary aim was to determine sociodemographic and health-related risk factors for diagnosed common mental disorders (CMDs) among young refugees in Sweden.

**Methods:**

All young adult refugees from Iran, Somalia and Syria (*n* = 7192), who were residents in Sweden in 2009, were followed from 2010 to 2013 regarding diagnosed CMDs. Cox regression models were used to compute hazard ratios (HRs) of CMDs with 95% confidence intervals (CIs).

**Results:**

Those arriving as unaccompanied refugee minors had a lower risk of being diagnosed with CMDs (HR: 0.7; 95%CI: 0.6–0.9) than those arriving as accompanied refugee minors. A higher risk of being diagnosed with CMDs was also found in female refugees (HR: 1.3; 95%CI: 1.1–1.5) compared with male refugees. In addition, individuals with a low (HR: 1.7; 95%CI: 1.3–2.3) or a medium (HR: 1.4; 95%CI: 1.1–1.8) educational level were found to have a higher risk of being diagnosed with CMDs compared with individuals with a high educational level. Refugees from Iran (HR: 2.3; 95%CI: 1.8–2.9) had a higher risk of a diagnosis of a CMD than refugees from Somalia. Moreover, refugees with a diagnosis of a mental disorder other than a CMD (HR: 4.2; 95%CI: 2.8–6.1), digestive (HR: 1.5; 95%CI: 1.0–2.2) or musculoskeletal diseases (HR: 1.5; 95%CI: 1.0–2.2) had a higher risk of being diagnosed with subsequent CMDs, compared with those with no such disorders.

**Conclusions:**

Pre-existing diagnoses of mental disorders other than CMDs, and digestive and musculoskeletal disorders should be carefully considered in clinical assessments to initiate early interventions to prevent CMDs.

## Introduction

According to the United Nations High Commissioner for Refugees (UNHCR), 68.5 million individuals were forcibly displaced in 2017 as a result of persecution, conflict, violence or human rights violations.^1^ Approximately 1 billion individuals are currently displaced from their initial homes.^2^ During the last decades, Sweden has received more refugees per capita than most other countries in Europe. More than half of the refugees in 2017 were children below 18 years and in 2015 alone, Sweden received 35 369 unaccompanied refugee minors.^1^^,^^3^ Therefore, this shift in refugee demographics in Sweden puts, even more, emphasis on the urgency to longitudinally elucidate the health status of those who migrated as young refugees.^4^ The largest refugee groups in Sweden are individuals from Iran, Somalia or Syria, and this population-based register study will elucidate risk factors for Common mental disorders (CMDs), that is depression, anxiety and stress-related disorders, in a cohort of young adult refugees. In addition, it will provide information regarding which risk factors may be considered for targeted interventions aiming to reduce the number of CMDs and thereby enhance social integration.

Refugees in high-income countries have, in general, been found to have poorer mental health than non-refugees, even when compared with non-refugee immigrants originating from the same country.^5^^,^^6^ Their mental health problems might be further aggravated after resettlement.^7^ Studies that longitudinally can elucidate the social integration and health development among this group are, therefore, warranted.^8^ Mental disorders are accountable for a large global burden of disease and they cause more years lived with disability than any other disorder.^9^ CMDs accounted for around three-quarters of all types of mental disorders, and they usually have an early age of onset and are often persistent and reoccurring.^10^^,^^11^ Young adults with CMDs are also, to a high extent, excluded from the labour market by being granted disability pension or by having long periods of both unemployment and sickness absence.^12^^,^^13^ Labour market marginalisation has been reported to further worsen mental health problems.^10^^,^^14^ Also, differences regarding mental health between refugees from Iran and Somalia have been shown in a study from the Netherlands,^15^ so the country of birth is an important factor to consider when studying mental health among refugees. Furthermore, studies have shown that a high proportion of unaccompanied refugee children suffer from post-traumatic stress disorder (PTSD).^16^ Unaccompanied refugee children are reported to be especially vulnerable, as they do not have their parents to help them cope with the situation in the host country.

Sociodemographic factors, including sex, educational level, family composition and type of living area, have been reported to be of importance for the development of CMDs. Also, the region of birth and duration of residence in the host country are important for the development and diagnostics of CMDs.^12^^,^^17^ Likewise, both somatic and other psychiatric comorbidities seem to affect the occurrence of CMDs.^18^^,^^19^ However, there are currently very few longitudinal studies regarding CMDs among young adult refugees. Identification of potentially modifiable risk factors of CMDs among young refugees is important in preventing future adverse health-related outcomes. The aim of this study was, therefore, to identify sociodemographic and health-related risk factors for diagnosed CMDs among young refugees from Iran, Somalia and Syria in Sweden.

## Material and methods

### Study population

The cohort was defined as all refugees, aged 18–25, from Iran, Somalia and Syria who were residents in Sweden on 31 December 2009 (*n* = 7464). Refugees with a baseline CMD (*n* = 272) were excluded, and the final study population included 7192 individuals. They were followed from 2010 until 2013 regarding CMDs. The definition of ‘refugees’ refers to the Geneva Convention on refugees as individuals: ‘… who are unable or unwilling to return to their country of birth owing to a well-founded fear of being persecuted for reasons of race, religion, nationality, membership of a particular social group or political opinion’,^20^ as well as humanitarian refugees and those who are ‘in need of protection’.^21^

A cohort of refugees who came to Sweden as minors (*n* = 4791), including unaccompanied refugee minors (*n* = 1991) and accompanied refugee minors (*n* = 2800), was analysed separately. The term ‘unaccompanied refugee minors’ refers to refugee minors who came to Sweden under the age of 19 without being accompanied by either of their parents. Refugee minors arriving in Sweden under the age of 19 with at least one parent were referred to as accompanied refugee minors.

### Type of data and data sources

Data on sociodemographic and health-related factors were collected from six nationwide Swedish registers, held by three government agencies: (i) statistics Sweden: *longitudinal integration database for health insurance and labour market studies*, for data on sex, age, educational level, family composition, type of living area, country of birth and year of immigration (2009); *Longitudinal database for integration studies,* for data on the reason for settlement in Sweden (2009), (ii) Social Insurance Agency: *microdata for analyses of social insurance register,* for data on disability pension (2009), (iii) National Board of Health and Welfare: *national patient register*, for data on dates and diagnoses of inpatient and specialized outpatient healthcare (2009–13); *Cause of death register*, for data on date of death (2009–13) and *Prescribed drug register* for data on prescriptions of dispensed antidepressant and diabetes medication (2009–13).

### Variables

#### Exposure

The exposure variables were divided into two groups:

1) Sociodemographic factors were measured on 31 December 2009 and included: sex [men (ref.) women], country of birth [Iran, Somalia (ref.) and Syria], refugee minor status [accompanied minor (ref.) unaccompanied minor], duration of residence in Sweden [0–5 years (ref.), 6–10 years and >10 years], type of living area [big cities (ref.), medium-sized cities and small cities/villages], educational level [low (0–9 years), medium (10–12 years) and high (>12 years) (ref.), missing information following Statistics Sweden’s suggested classification^22^], family composition [married/living with a partner without children living at home, married/living with a partner with children living at home, single/divorced/separated/widowed without children living at home (ref.), single/divorced/separated/widowed with children living at home)].
2) Health-related factors were measured during 2009 and included disability pension [no disability pension [ref.] and granted disability pension]. In Sweden, a temporary disability pension is given to young adults between 19 and 29 years of age with functional impairment due to illness or an injury that lowered work capacity by >25% or if upper secondary schooling is not finished at the age of 19 years due to illness. All other health conditions were measured as a record of a visit to either inpatient or specialized outpatient healthcare according to the International Classification of Disorders-version 10 (ICD-10). In some cases, data on prescriptions of drugs were also included, obtained from the prescribed drug register according to the Anatomical Therapeutic Chemical Classification system (ATC). These cases include: CMDs (ICD-10: F32-33, F40-43 and ATC code: N06A), mental disorders other than CMDs (ICD-10: F00-31, F34-39 and F44-99), diabetes mellitus (ICD-10: E10-E14 and ATC code A10), circulatory system diseases (ICD-10: I00-I99), digestive system diseases (ICD-10: K00-K93), infectious and parasitic diseases (ICD-10: A00-B99), musculoskeletal system diseases (ICD-10: M00-M99), neoplasm (ICD-10: C00-D48), nervous system diseases (ICD-10: G00-G99), respiratory system diseases (ICD10: J00-J99) and other somatic disorders (ICD-10: E00-E09, E15-E90, H01-H99, L00–99 and N00-T99).

#### Outcome

CMDs during the follow-up period were defined as either a diagnosis from inpatient or specialized outpatient healthcare of depression (ICD-10: F32-F33), anxiety disorders (ICD-10: F40-F42) and stress-related disorders, including PTSD (ICD-10: F43) or a prescription of antidepressant medication (ATC: N06A).

### Statistical analysis

After testing that the proportional hazard assumption was met, hazard ratios (HRs) with 95% confidence intervals (CIs) were calculated by Cox regression models using SAS statistical software, version 9.4 (SAS Institute Inc., Cary, NC). Both crude models and multivariate models were conducted. The multivariate analyses were adjusted in two steps: (1) sociodemographic factors (Model 1) and (2) sociodemographic and health-related factors (Model 2). Censoring in the analyses was made for death, emigration or end of follow-up, whichever came first. In addition, we considered the possibility of collinearity, but the tests of the variance inflation factor showed that there was no strong connection between any of the independent variables. To rule out potential differences between categories of refugees included in the study, i.e. refugees that were granted a residence permit due to the Geneva Convention, on humanitarian grounds, or due to the need for protection, a sensitivity analysis was performed. The sensitivity analysis showed that the outcomes were similar for all three groups of refugees.

## Results

### Characteristics of the study population

There was about an equal number of men and women in the study cohort (Table 1). Regarding education, half of the study population had either a medium or high educational level; however, information on the educational level was missing for one-fifth of the study population. A vast majority of the study population (87%) were singles without children living at home and most individuals lived in big cities (49%). The largest group was from Somalia (48%), followed by refugees from Iran (38%) and Syria (14%). Almost half of the study population had lived in Sweden for > 10 years, and around two-thirds of them came to Sweden as refugee minors, either as unaccompanied minors (42%) or accompanied minors (58%).

**Table 1:** Characteristics of all refugees from Iran, Somalia and Syria without a baseline CMD, aged 18–25 years who were resident in Sweden in 2009 (*n* = 7192)

	*n* (%)
**Sociodemographic risk factors** ^**a**^	
***Sex***	
Men	3589 (49.9%)
Women	3603 (50.1%)
***Educational level***	
Low (0–9 years)	2101 (29.2%)
Medium (10–12 years)	2384 (33.1%)
High (>12 years)	1243 (17.3%)
Missing information	1464 (20.4%)
***Family composition*** ^**b**^	
Married/partnership no children at home	258 (3.6%)
Married/partnership with children at home	371 (5.1%)
Single^c^, no children at home	6219 (86.5%)
Single^c^ with children at home	344 (4.8%)
***Type of living area***	
Big cities	3493 (48.6%)
Medium-sized cities	2635 (36.6%)
Small cities/villages	1064 (14.8%)
***Country of birth***	
Somalia	3465 (48.2%)
Iran	2746 (38.2%)
Syria	981 (13.6%)
***Duration of residence in Sweden***	
0–5 years	3255 (45.2%)
6–10 years	637 (8.9%)
>10 years	3300 (45.9%)
***Came to Sweden as a refugee minor***	
Yes, unaccompanied by parent(s)	1991 (27.7%)
Yes, accompanied by parent(s)	2800 (38.9%)
No	2401 (33.4%)
**Health-related risk factors** ^**d**^	
*Disability pension*	88 (1.2%)
*Other mental disorders*	88 (1.2%)
*Diabetes mellitus*	26 (0.4%)
*Circulatory system diseases*	35 (0.5%)
*Digestive system diseases*	187 (2.6%)
*Infectious and parasitic diseases*	291 (4.1%)
*Musculoskeletal system diseases*	197 (2.7%)
*Neoplasm*	52 (0.7%)
*Nervous system diseases*	78 (1.1%)
*Respiratory system diseases*	184 (2.6%)
*Other somatic disorders*	1648 (22.9%)
**Common mental disorders** ^**e**^ **during follow-up**	**562 (7.8%)**
Inpatient healthcare	32 (0.4%)
Specialized outpatient healthcare	178 (2.5%)
Antidepressant prescription	352 (4.9%)

^a^All sociodemographic factors were measured on 31 December 2009.

^b^Married/partnership or single with or without children living at home.

^c^Single/divorced/separated/widowed.

^d^Other mental disorders included both inpatient and specialized outpatient healthcare (ICD-10: F00-31, F34-39 and F44-99). The following nine subgroups of somatic disorders included both inpatient and specialized outpatient healthcare due to: diabetes mellitus (ICD-10: E10-E14; ATC code: A10), circulatory system diseases (ICD-10: I00-I99), digestive system diseases (ICD-10: K00-K93), infectious and parasitic diseases (ICD-10: A00-B99), musculoskeletal system diseases (ICD-10: M00-M99), neoplasm (ICD-10: C00-D48), nervous system diseases (ICD-10: G00-G99), respiratory system diseases (ICD10: J00-J99) and other somatic disorders (ICD-10: E00-E09, E15-E90, H01 - H99, L00-99 and N00-T99). All health-related factors were measured in 2009.

^e^Common mental disorders included both inpatient and specialized outpatient healthcare (according to the ICD-10: F32-33, F40-43), as well as prescriptions for antidepressants taken from prescribed drug register [according to the ATC Classification code: N06A].

**Table 2:** HRs with 95% CIs for sociodemographic risk factors for subsequent CMDs among refugees aged 18–25 years from Iran, Somalia and Syria without a baseline CMD, who were resident in Sweden in 2009 (*n* = 7192)

		**Crude Model**	**Model 1** ^**a**^	**Model 2** ^**b**^
	***n* (%)** ^**c**^	**HR (95% CI)**	**HR (95% CI)**	**HR (95% CI)**
***Sex***				
Men	302 (8.4)	1	1	1
Women	260 (7.2)	1.17 (0.99–1.38)	**1.34 (1.13–1.59)**	**1.27 (1.07–1.51)**
***Educational level***				
Low (0–9 years)	182 (8.6)	1.17 (0.91–1.50)	**1.91 (1.43–2.55)**	**1.72 (1.28–2.29)**
Medium (10–12 years)	212 (8.9)	1.21 (0.95–1.55)	**1.45 (1.13–1.86)**	**1.38 (1.08–1.77)**
High (>12 years)	93 (7.5)	1	1	1
Missing	75 (5.1)	**0.68 (0.50–0.92)**	1.31 (0.90–1.90)	1.20 (0.82–1.76)
***Family composition*** ^**d**^				
Married/partnership, no children	14 (5.4)	1.08 (0.75–1.54)	1.17 (0.81–1.68)	1.01 (0.70–1.47)
Married/partnership with children	32 (8.6)	0.66 (0.39–1.12)	0.65 (0.38–1.12)	0.62 (0.36–1.06)
Single^e^, no children	499 (8.0)	1	1	1
Single^e^ with children	17 (4.9)	**0.61 (0.38–0.99)**	0.76 (0.46–1.27)	0.64 (0.38–1.06)
***Type of living area***				
Big cities	284 (8.1)	1	1	1
Medium-sized cities	204 (7.7)	0.95 (0.79–1.13)	1.09 (0.91–1.31)	1.08 (0.90–1.29)
Small cities/villages	74 (6.9)	0.85 (0.66–1.10)	1.10 (0.84–1.45)	1.13 (0.86–1.48)
***Country of birth***				
Refugee, Somalia	182 (5.2)	1	1	1
Refugee, Iran	322 (11.7)	**2.32 (1.93–2.78)**	**2.35 (1.86–2.97)**	**2.33 (1.84–2.94)**
Refugee, Syria	58 (5.9)	1.12 (0.84–1.51)	1.08 (0.78–1.51)	1.12 (0.81–1.56)
***Duration of residence in Sweden***				
0–5 years	207 (6.4)	1	1	1
6–10 years	50 (7.8)	1.25 (0.92–1.70)	0.82 (0.56–1.20)	0.89 (0.61–1.30)
>10 years	305 (9.2)	**1.48 (1.24–1.76)**	0.93 (0.67–1.29)	1.01 (0.73–1.40)

^a^ **Model 1**. Adjusted for sex, educational level, family composition, type of living area, country of birth, duration of residence in Sweden and refugee minor. All sociodemographic variables were measured on 31 December 2009.

^b^ **Model 2**. Model 1 and additionally adjusted for disability pension, other mental disorders, diabetes mellitus, circulatory system diseases, digestive system diseases, infectious and parasitic diseases, musculoskeletal system diseases, neoplasm, nervous system diseases, respiratory system diseases and other somatic disorders. All health-related variables were measured in 2009.

^c^Number and percentage of refugees with the outcome common mental disorder during follow-up 2010–13.

^d^Married/partnership or single with or without children living at home.

^e^Single/divorced/separated/widowed. Bold numbers indicate significant HRs.

**Table 3:** HRs with 95% CIs for sociodemographic and health-related factors for subsequent CMDs among refugees aged 18–25 years from Iran, Somalia and Syria without a baseline CMD, who were resident in Sweden in 2009 and came to Sweden as refugee minors (*n* = 4791)

		**Crude Model**	**Model 1** ^**a**^	**Model 2** ^**b**^
	**Outcome *n* (%)** ^**c**^	**HR (95% CI)**	**HR (95% CI)**	**HR (95% CI)**
***Refugee minors***				
Accompanied	271 (9.7)	1	1	1
Unaccompanied^d^	152 (7.6)	**0.72 (0.60–0.85)**	**0.70 (0.56–0.88)**	**0.74 (0.59–0.93)**

^a^ **Model 1**. Adjusted for sex, educational level, family composition, type of living area, country of birth, duration of residence in Sweden and refugee minor. All sociodemographic variables were measured on 31 December 2009.

^b^ **Model 2**. Model 1 and additionally adjusted for disability pension, other mental disorders, diabetes mellitus, circulatory system diseases, digestive system diseases, infectious and parasitic diseases, musculoskeletal system diseases, neoplasm, nervous system diseases, respiratory system diseases and other somatic disorders. All health-related variables were measured in 2009.

^c^Number and percentage of refugees with the outcome common mental disorder during follow-up 2010–13.

^d^An unaccompanied minor was defined as coming to Sweden under the age of 19 years with no parents. Bold numbers indicate significant HRs.

**Table 4:** HRs with 95% CIs for health-related risk factors^a^ for subsequent CMDs^b^ among refugees aged 18–25 years from Iran, Somalia and Syria without a baseline CMD, who were resident in Sweden in 2009 (*n* = 7192)

		**Crude Model**	**Model 1** ^**c**^	**Model 2** ^**d**^
	***n* (%)** ^**e**^	**HR (95% CI)**	**HR (95% CI)**	**HR (95% CI)**
***Disability pension***				
No disability pension in 2009	548 (7.7)	1	1	1
Disability pension in 2009	14 (15.9)	**2.17 (1.28–3.68)**	**1.73 (1.00–3.00)**	0.95 (0.53–1.69)
***Diabetes mellitus***				
No	559 (7.8)	-	-	-
Yes	< 10	-	-	-
***Circulatory system diseases***				
No	560 (7.8)	-	-	-
Yes	< 10	-	-	-
***Digestive system diseases***				
No	534 (7.6)	1	1	1
Yes	28 (15.0)	**2.05 (1.40–3.00)**	**2.06 (1.41–3.02)**	**1.50 (1.01–2.23)**
***Infectious and parasitic diseases***				
No	524 (7.6)	1	1	1
Yes	38 (13.1)	**1.77 (1.27–2.46)**	**1.78 (1.28–2.47)**	1.39 (0.99–1.95)
***Musculoskeletal system diseases***				
No	533 (7.6)	1	1	1
Yes	29 (14.7)	**2.00 (1.38–2.91)**	**1.87 (1.28–2.72)**	**1.48 (1.01–2.17)**
***Neoplasm***				
No	557 (7.8)	-	-	-
Yes	< 10	-	-	-
***Nervous system diseases***				
No	548 (7.7)	1	1	1
Yes	14 (17.9)	**2.43 (1.43–4.12)**	**2.48 (1.46–4.24)**	1.60 (0.92–2.78)
***Respiratory system diseases***				
No	542 (7.7)	1	1	1
Yes	20 (10.9)	1.42 (0.91–2.22)	1.34 (0.86–2.10)	1.08 (0.68–1.69)
***Other mental disorders***				
No	530 (7.5)	1	1	1
Yes	32 (36.4)	**5.94 (4.16–8.49)**	**5.35 (3.72–7.69)**	**4.17 (2.84–6.14)**
***Other somatic disorders***				
No	366 (6.6)	1	1	1
Yes	196 (11.9)	**1.85 (1.55–2.20)**	**1.85 (1.55–2.21)**	**1.60 (1.33–1.94)**

^a^Other mental disorders included both inpatient and specialized outpatient healthcare (ICD-10: F00-31, F34-39 and F44-99). The following nine subgroups of somatic disorders included both inpatient and specialized outpatient healthcare due to: diabetes mellitus (ICD-10: E10-E14; ATC code A10), circulatory system diseases (ICD-10: I00-I99), digestive system diseases (ICD-10: K00-K93), infectious and parasitic diseases (ICD-10: A00-B99), musculoskeletal system diseases (ICD-10: M00-M99), neoplasm (ICD-10: C00-D48), nervous system diseases (ICD-10: G00-G99), respiratory system diseases (ICD10: J00-J99) and other somatic disorders (ICD-10: E00-E09, E15-E90, H01-H99, L00-99 and N00-T99). All health-related factors were measured in 2009.

^b^CMDs included both inpatient and specialized outpatient healthcare (according to the ICD-10: F32-33, F40-43), as well as prescriptions for antidepressants taken from prescribed drug register [according to the ATC Classification code: N06A].

^c^ **Model 1**. Adjusted for sex, educational level, family composition, type of living area, country of birth, duration of residence in Sweden and refugee minor. All sociodemographic variables were measured on 31 December 2009.

^d^ **Model 2**. Model 1 and additionally adjusted for disability pension, other mental disorders, diabetes mellitus, circulatory system diseases, digestive system diseases, infectious and parasitic diseases, musculoskeletal system diseases, neoplasm, nervous system diseases, respiratory system diseases and other somatic disorders. All health-related variables were measured in 2009.

^e^Number and percentage of refugees with the outcome common mental disorder during follow-up 2010–13. Bold numbers indicate significant HRs.

### Sociodemographic risk factors

Women in the multivariate model had a slightly higher risk of CMDs compared with men (HR: 1.3, Table 2). Refugees with both low (HR: 1.7) and medium (HR: 1.4) educational level in the multivariate model had higher risk of CMDs than refugees with high educational level. Young refugees from Iran had, both in the crude and the multivariate models, a higher risk of CMDs than refugees from Somalia (HR: 2.3). Refugees who arrived in Sweden as unaccompanied refugee minors were less likely to have CMDs compared with the accompanied refugee minors, shown both in the crude model (HR: 0.7, Table 3) and in the multivariate model (HR: 0.7).

### Health-related factors

Refugees who had disability pension at the baseline had a significantly higher risk of CMDs compared with those without disability pension (HR: 2.2) in the crude model, this was, however, not found in the multivariate model (HR: 1.0, Table 4). Refugees with a baseline diagnosis of a mental disorder other than CMDs had a 6-fold higher risk for CMDs in the crude model (HR: 6.0), and more than a 4-fold higher risk of CMDs in the multivariate model (HR: 4.2), compared with those without other mental disorders. Refugees with digestive system disorders (HR: 2.1), infectious and parasitic diseases (HR: 1.8), musculoskeletal disorders (HR: 2.0), nervous system diseases (HR: 2.4) and the group of ‘other somatic disorders’ (HR: 1.9) had a higher risk of CMDs in the crude model. However, in the final adjusted model, the association existed only for digestive system diseases (HR: 1.5), musculoskeletal system diseases (HR: 1.5) and the group of ‘other somatic disorders’ (HR: 1.6).

## Discussion

### Main findings of this study

Women had a higher risk of CMDs compared with men. Refugees with low and medium educational levels had a higher risk of CMDs compared with refugees with high educational levels. Young refugees from Iran were more likely to have CMDs than young refugees from Somalia. Those who came to Sweden as unaccompanied refugee minors as young adults had a slightly lower risk for CMDs compared with those who came to Sweden as accompanied refugee minors. A previous diagnosis of a mental disorder other than a CMD significantly increased the individuals’ risk for CMDs, and so did digestive system diseases and musculoskeletal system diseases.

### What is already known on this topic

#### Sociodemographic risk factors for CMDs

Refugee women from Iran, Somalia and Syria had a higher risk of CMDs than refugee men. This is also consistent with other studies, which reports that women have a higher risk of mental illness compared with men among Syrian refugees^23^ and in a subpopulation of refugees who have experienced trauma.^24^ A possible reason for this could be the higher level of vulnerability among women during the migration process than among men.^25^ This is mainly attributed to being the principal caregiver for the children and to being less integrated into the host country compared with refugee men.^25^ Moreover, common mental disorders are shown to be more prevalent in women than men—even in other populations.^26^

Young adult refugees with low and medium educational levels had a higher risk of CMDs compared with those with high educational levels. This has also been reported by other studies, both in the general population and among migrants in general.^27^^,^^28^ A high educational level usually implies a higher awareness of one’s state of health, which means that one is more likely to notice the symptoms of CMDs and to seek healthcare and treatment, and as a result, this information will be found in the registers.^29^ However, in a study on Syrian refugees aged 18–64 in Sweden, the prevalence of mental disorders did not differ concerning educational level.^23^ In a worldwide meta-analysis, the opposite association was found, i.e. those with a high educational level had the highest risk of developing mental disorders.^30^ As a high educational level mostly equals to high socioeconomic status, loss of status is likely to lead to poorer health.^30^^,^^31^ Low educational level is associated with labour market marginalisation, i.e. hardship in obtaining and retaining a job.^12^^,^^29^^,^^32^ Long periods of being outside the labour market may further lead to deteriorating health, especially CMDs.^14^^,^^33^ Here, given the right prerequisites by stakeholders, such as employers and employment agencies, early working age may offer better chances to succeed in the labour market.

Young refugees from Iran had a more than 2-fold higher risk of subsequent CMDs compared with young Somalian refugees. Iranian immigrants in Sweden have been shown to have relatively high educational levels, but have also faced difficulties in transferring their education and credentials from Iran into the Swedish context.^34^ According to the discussion above, the loss of socioeconomic status might have been especially cumbersome among refugees from Iran. Moreover, it is important to point out that the findings for Iranian refugees were compared with those from Somalia. A study from Norway reported that migrants from Somalia, to a higher extent, do not seek healthcare due to stigmatization of mental disorders, especially psychiatric healthcare, but instead consult family, friends and their community.^35^ A Dutch study has also reported that refugees from Iran had a much higher probability of having both depression and PTSD compared with Somalian refugees.^15^ The high risk among Iranian refugees found in our study is consistent with previous studies and may be due related to the aforementioned loss of socioeconomic status; in contrast, Somalian refugees may not necessarily have a lower risk of CMDs, but a lower risk of being diagnosed based on healthcare engagement practices. Somalian refugees have also a higher probability of being outside the labour market compared with most other refugee groups.^5^ Such cultural differences among refugees from different countries must be addressed by the employment agencies and other stakeholders to give adequate support for labour market integration for refugees.

Refugees that arrived in Sweden as unaccompanied refugee minors had a lower risk for CMDs in young adult age compared with those who arrived in Sweden as accompanied refugee minors. It is more intuitive to expect the other way around, i.e. that the accompanied refugee minors are more protected due to their possible support from their parents. Thus, unaccompanied refugee minors have in other studies been reported to have a higher risk of inpatient healthcare due to mental disorders compared with accompanied refugee minors.^36^ There could be several explanations for our findings. One plausible assumption could be a selection process, i.e. those who successfully reached Sweden despite being unaccompanied by parents indicated that they had been more resilient, to begin with, both physically and mentally, and that they were, therefore, better adjusted to their new life in Sweden.^37^ Studies have reported that unaccompanied and separated asylum-seeking children are recognized as a vulnerable group with special needs in the asylum procedure.^38^^,^^39^ Another explanation could hence be that because refugees who arrived as unaccompanied minors were believed to be especially vulnerable— they had received more attention and care.^40^

#### Health-related risk factors for CMDs

Those who had a comorbid mental disorder (other than a CMD) during the baseline year had the most pronounced risk of having CMDs in the follow-up period. Mental disorders are known to usually have an early age of onset and are associated with comorbidity, as a result, the risk of CMDs is higher among those who have previously been diagnosed with a mental disorder other than CMD.^12^ Furthermore, the results showed that digestive system diseases and musculoskeletal system diseases at baseline were risk factors for later CMDs. These disorders are known to be comorbid with CMDs.^18^^,^^41^ Individuals with CMDs may have mistaken their symptoms for digestive or musculoskeletal diseases, and consequently, CMDs were overlooked as the real underlying cause for those symptoms. Similarly, musculoskeletal system diseases could also be psychosomatic. Due to the fear of stigma and discrimination, many immigrants do not seek healthcare for mental disorders.^35^^,^^42^ Here, CMDs might be clinically manifested as somatic complaints among young refugees, which in turn might lead to physicians without adequate training in transcultural psychiatry not adequately diagnosing and treating the underlying mental disorder.^43^

#### What this study adds

By registering linkage^22^^,^^44^^,^^45^ in a population-based setting, we found several risk factors for the development of CMDs among refugees from Iran, Somalia and Syria in Sweden. One of the most interesting findings from the study was that those who arrived in Sweden as unaccompanied refugee minors had a lower risk for CMDs in young adult age compared with those who arrived in Sweden as accompanied refugee minors. We also found that there was a significantly higher risk for refugees from Iran to have a diagnosis of CMDs, which may be attributed to different barriers to seeking healthcare among some groups. Young adult refugee women had a higher risk of being diagnosed with a CMD than men, and educational level was of importance for the development of CMDs in young adult age.

#### Limitations of this study

One of the limitations of this study is that only refugees who had residence permits were included in this study. This means that the register data does not include asylum seekers who are living in Sweden, but who have not yet received a decision on their asylum application. This may lead to an underestimation of the risk of CMDs, as asylum seekers have been shown to have worse health status compared with refugees with residence permits.^37^ As mental disorders are highly stigmatized, especially among some refugee groups, there might be individuals with CMDs who did not seek medical care, which consequently led to an underestimation of the number of individuals with CMDs.^46^^,^^47^ Another limitation may arise from the definition of unaccompanied minors. In this study, unaccompanied minors were defined as those who came to Sweden without any of their parents accompanying them. However, in reality, minors could have arrived in Sweden with siblings or other relatives, which we could not identify. Refugees most recently coming to Sweden could not be included in this study due to lags in registers. The cohort was followed during 2010–13 and may, therefore, not represent the most recent situation among refugees in Sweden. For example, very few individuals fleeing from the Syrian war that started in 2011 were included in the study. Even if there were no major changes in the healthcare regulations affecting the diagnostics of CMDs, the inclusion of refugees fleeing from the Syrian war might have altered the composition of individuals included in the study.

## Conclusions

The study reveals that young refugee women and young refugees with a low educational level have a high risk of developing CMDs. Furthermore, the country of birth may be of importance for the probability of being diagnosed with a CMD. Another important finding is that accompanied refugee minors may be prone to a higher risk of CMDs. Moreover, the identified health-related risk factors, such as diagnoses of mental disorders other than CMDs, digestive system disorders and musculoskeletal system disorders should be carefully considered by healthcare providers when monitoring young refugees who seek healthcare. It is particularly important to watch out for potentially undiagnosed CMDs or CMDs disguised as somatic disorders.

## Funding

This work study was supported by the Swedish Research Council, grant number 2018-05783.

## Conflict of Interest statement

The authors declare that they have no conflict of interest.

## Ethics approval

Ethical approval of this study was given by the Regional Ethical Board in Stockholm. The Ethical board also waived the requirement of informed consent from the participants in this study.

## Data availability

The data in this study are available from Statistics Sweden. The data are not publicly available but can be accessed if ethical permission is received.

## Notes

**Lijun Yang**, Ms

**Ellenor Mittendorfer-Rutz**, Professor

**Ridwanul Amin,** PhD

**Magnus Helgesson**, Assistant Professor
